# Nicotinaldehyde, a Novel Precursor of NAD Biosynthesis, Abrogates the Anti-Cancer Activity of an NAD-Lowering Agent in Leukemia

**DOI:** 10.3390/cancers15030787

**Published:** 2023-01-27

**Authors:** Saki Matsumoto, Paulina Biniecka, Axel Bellotti, Michel A. Duchosal, Aimable Nahimana

**Affiliations:** 1 Central Laboratory of Hematology, Department of Medical Laboratory and Pathology, Lausanne University Hospital and University of Lausanne, Rue du Bugnon 27, 1011 Lausanne, Switzerland; 2 Service of Hematology, Department of Oncology, Lausanne University Hospital and University of Lausanne, Rue du Bugnon 46, 1011 Lausanne, Switzerland

**Keywords:** NAD, nicotinaldehyde, APO866, NAMPT inhibitor, acute myeloid leukemia

## Abstract

**Simple Summary:**

Cancer cells are reliant on a sufficient amount of nicotinamide adenine nucleotide (NAD) to sustain their altered metabolism and proliferation. Targeting NAD depletion with inhibitors of NAD biosynthesis has therefore emerged as a promising approach for cancer treatment. Growing evidence demonstrates the existence of multiple precursors and alternative pathways for NAD biosynthesis, and that many parameters that include gut microbiota may negatively affect the therapeutic efficacy of NAD lowering agents in cancer treatment. These findings raise the need to depict further the NAD biogenesis in mammalian cells in order to improve the efficacy of NAD targeting anti-cancer treatment. In the present study, we report the identification of nicotinaldehyde as a novel NAD precursor in leukemia cells. Our findings reveal the implication of a novel NAD metabolite in modulating the anti-cancer efficacy of an NAD-lowering agent and suggest potential strategies to enhance its therapeutic effect.

**Abstract:**

Targeting NAD depletion in cancer cells has emerged as an attractive therapeutic strategy for cancer treatment, based on the higher reliance of malignant vs. healthy cells on NAD to sustain their aberrant proliferation and altered metabolism. NAD depletion is exquisitely observed when NAMPT, a key enzyme for the biosynthesis of NAD, is inhibited. Growing evidence suggests that alternative NAD sources present in a tumor environment can bypass NAMPT and render its inhibition ineffective. Here, we report the identification of nicotinaldehyde as a novel precursor that can be used for NAD biosynthesis by human leukemia cells. Nicotinaldehyde supplementation replenishes the intracellular NAD level in leukemia cells treated with NAMPT inhibitor APO866 and prevents APO866-induced oxidative stress, mitochondrial dysfunction and ATP depletion. We show here that NAD biosynthesis from nicotinaldehyde depends on NAPRT and occurs via the Preiss–Handler pathway. The availability of nicotinaldehyde in a tumor environment fully blunts the antitumor activity of APO866 in vitro and in vivo. This is the first study to report the role of nicotinaldehyde in the NAD-targeted anti-cancer treatment, highlighting the importance of the tumor metabolic environment in modulating the efficacy of NAD-lowering cancer therapy.

## 1. Introduction

Nicotinamide adenine dinucleotide (NAD) is an essential metabolite in cell life, participating in myriads of cellular fundamental processes. NAD (referring to both the oxidized NAD^+^ and reduced NADH forms) has a major role in the energy metabolism by serving as a co-factor to catalyze redox reactions involved in several metabolic pathways, including glycolysis, TCA cycle and oxidative phosphorylation. It also serves as a substrate for enzymes such as sirtuins, poly-ADP-ribose polymerases (PARPs), and cyclic ADP ribose synthases (such as CD38 and CD157), and therefore plays essential roles in gene expression, DNA damage repair, stress response signaling, or regulation of apoptosis [1]. A sustained production of NAD and the maintenance of its sufficient level are crucial for the survival and proliferation of both healthy and malignant cells. Due to their rapid proliferation, cancer cells are notably more exposed to environmental and intrinsic threats, such as metabolic stresses and DNA synthesis needs or DNA damaging insults. Moreover, they are in continuous need of producing energy and rely on a sustained biosynthesis of NAD [2]. For these reasons, targeting NAD depletion in malignant cells has emerged as an attractive therapeutic strategy for anti-cancer treatment.

Mammalian cells can produce NAD through three major pathways from different NAD precursors [3]: (i) nicotinamide (NAM) or its related riboside form (NR), through the “salvage” pathway; (ii) nicotinic acid (NA) or its related riboside form (NAR), through the “Preiss–Handler” pathway; or (iii) tryptophan through the so-called “de novo” pathway. NAM and NA are the most important precursors and are widely used as NAD precursors in mammals [4,5,6,7]. Within the salvage pathway, nicotinamide phosphoribosyltransferase (NAMPT) acts as the rate-limiting enzyme and converts NAM to NMN, which is then converted to NAD by NMN adenylyltransferase (NMNAT). This pathway is of major importance as it enables the recycling of NAM, the end product of NAD utilization by NAD-consuming enzymes, back to NAD.

In line with this notion, the inhibition of NAMPT with small molecule inhibitors has been extensively studied as a promising anti-cancer treatment. Several inhibitors of NAMPT have been developed to date, among which APO866 (also known as FK866 or Daporinad) was the first reported and the most extensively studied [8,9,10]. In several mouse xenograft models, NAMPT inhibitors induced the inhibitory effect on tumor initiation and progression, leading to the prolongation of overall survival of the animals [11,12]. These promising preclinical results led to their assessment in clinical trials, however, none of them succeeded in showing an objective and consistent tumor response in patients [13,14]. This implies a strong need to optimize the use of NAMPT inhibitors in clinical settings. In the attempt to explain the lack of efficacy in clinical trials, recent studies demonstrated that gut microbiota and metabolites available in the tumor environment could greatly contribute to counteracting the antitumor activity of NAMPT inhibitors. This was operating through the activation of NAD biosynthetic pathways alternative to that blocked by NAMPT inhibitors, enabling the replenishment of intracellular NAD [15,16]. These reports highlight the complexity of the inter-conversion of substrates for NAD biosynthesis and the importance of controlling the tumor metabolic environment for optimizing the therapeutic efficacy of NAMPT inhibitors.

Nicotinaldehyde is a natural compound found in plants and bacteria, and it is used as an intermediate for molecule synthesis in the pharmaceutical and agronomical industries. It can also be contained in foods of plant origin such as honey [17]. To date, only a few studies have investigated the role of nicotinaldehyde in NAD metabolism in humans.

In this study, we report the identification of nicotinaldehyde as a novel NAD precursor that can be used for NAD generation by leukemia cells. Notably, we show that nicotinaldehyde supplementation abrogates the antitumor properties of NAMPT inhibition with APO866 both in vitro and in vivo, through an enzymatic conversion to NA and activation of an alternative NAPRT-dependent biosynthesis of NAD. Of interest, we provide the first description of the potential negative impact of nicotinaldehyde on the anti-leukemia activities of NAMPT inhibitors.

## 2. Materials and Methods

### 2.1. Cell Lines, Primary Cells and Culture Conditions

Six hematological cancer cell lines were purchased from DSMZ (German Collection of Microorganisms and Cell Cultures) or ATCC and include Jurkat (T-acute lymphoblastic leukemia); ML2, SKM-1 and NOMO-1 (acute myeloid leukemia); and Namalwa and Ramos (Burkitt lymphoma). The molecular profiles are available at DSMZ or ATCC. CD38 expression profiles are provided in Figure S1. ML2 NAPRT KO cells were generated by the CRISPR genome editing method as described previously [16]. All cells were cultured in RPMI (Invitrogen AG, 61870-01, Life Technologies, Zug, Switzerland) supplemented with 10% heat inactivated fetal calf serum (FCS, Amimed, 2-01F30-I) and 1% penicillin/streptomycin (PS) (Amimed, 4-01F00-H, BioConcept, Allschwil, Switzerland) at 37 °C in a humidified atmosphere of 95% air and 5% CO_2_.

### 2.2. Flow Cytometry

To evaluate the cytotoxic effect of APO866 (kindly provided by TopoTarget, Switzerland) alone or in combination with nicotinaldehyde (Sigma, P62208, Sigma-Aldrich Chemie GmbH, Buchs, Switzerland), cancer cells were incubated without or with 10 nM (if not specified otherwise) of APO866 in the absence or presence of 10 μM of nicotinaldehyde and stained with annexin-V (eBioscience, BMS306FI/300, Life Technologies) and 7-aminoactinomycin D (7AAD, A07704, Beckman Coulter, Nyon, Switzerland) as described by the manufacturer and analyzed using flow cytometry. Dead cells were identified as 7AAD^+^ and early apoptotic cells as annexin^+^ 7AAD^−^. Live cell number was counted with CountBright™ Plus Absolute Counting Beads (Invitrogen, C36995). Mitochondrial membrane potential (MMP) was evaluated with Tetramethylrhodamine methyl ester (TMRM) staining (Invitrogen, T668, Thermo Fisher Scientific, Reinach, Switzerland). After a 96 h treatment with APO866 and/or nicotinaldehyde, cells were washed with warm PBS and resuspended in PBS containing 100 nM TMRM dye for 30 min at 37 °C, and the fluorescence level was measured by flow cytometry. The levels of intracellular ROS, namely mitochondrial and cytosolic superoxide anion radicals O_2_^−^ and total H_2_O_2_, were assessed using specific fluorescent probes, Mitosox (Molecular Probes, M36008, Life Technologies), DHE (Marker Gene Technologies, MGT-M1241-M010, Abcam, Amsterdam, Netherlands) and carboxy-H2DCFDA (Molecular Probes, C-400, Life Technologies), respectively. After a 96 h treatment with APO866 and/or nicotinaldehyde, cells were washed with PBS (or DPBS for carboxy-H2DCFDA) and resuspended in PBS containing 2.5 µM, 10 µM and 20 µM of specific probes, respectively, for 30 min at 37 °C, and the fluorescence level was assessed by flow cytometry.

### 2.3. Determination of Intracellular NAD and ATP Content by Biochemical Assay

Moreover, 3 × 10^5^ cells were seeded in 24-well plates in the presence or absence of APO866 or nicotinaldehyde supplementation. At 96 h of incubation at 37 °C, cells were harvested by centrifugation at 12,000 rpm at 4 °C for 5 min followed by a wash with cold PBS and suspension in lysis buffer (20 mM NaHCO_3_, 100 mM Na_2_CO_3_). Total intracellular NAD content was measured using a colorimetric cycling assay. Briefly, cell lysates were incubated in the presence of Phenazine Ethosulfate (PES), 3-(4,5-Dimethylthiazol-2-yl)-2,5-Diphenyl-tetrazolium Bromide (MTT), Alcohol Dehydrogenase (ADH) and ethanol. When ADH oxidizes ethanol, NAD^+^ is reduced to NADH, which further allows the reduction/oxidation of PES and MTT. The resulted formation of reduced MTTH can be monitored as an increase of absorbance at 570 nm. The rate of MTTH appearance is proportional to the concentration of NAD(H) present in the assay mixture. The values were normalized by protein concentrations determined with BCA protein assay. Total ATP content was measured using the ATP determination Kit (Life Technologies, A22066) according to the manufacturer’s instructions.

### 2.4. Quantification of NAD Metabolome Using LC-MS/MS

For NAD-related metabolites quantification, cells were incubated at 37 °C for 96 h in the presence or absence of APO866 (10 nM), without or with nicotinaldehyde supplementation (10 µM) in the RPMI culture medium containing 10% FCS and 1% PS. After centrifugation at 14,000× *g* for 15 min, the conditioned medium (or the supernatant) and the cell pellets were collected separately. RPMI medium (without cells) was collected similarly after supplementation with nicotinaldehyde (10 µM) and incubation at 37 °C for 96 h. NAD-related metabolites levels in the obtained samples were evaluated by LC-MS/MS as described previously [16].

### 2.5. Evaluation of In Vivo Antitumor Efficacy of APO866 in Mouse Xenograft Models

Severe combined immune deficiency (SCID) mice (Iffa Credo, L’Arbresle, France) were housed in micro-isolator cages in a specific pathogen-free room within the animal facilities at the University Hospital of Lausanne. Animals were allowed to acclimatize to their new environment for 1 week prior to use. All animal experiments were conducted according to the respective institutional regulations after the approval of the animal ethics committee of the University of Lausanne. Mice were transplanted subcutaneously into the right flank with 1 × 10^7^ ML2 human AML cells. Once the tumor reached a volume of 100 mm^3^, mice were subdivided into groups of treatments (vehicle, nicotinaldehyde, APO866, nicotinaldehyde and APO866). APO866 (15 mg/kg body weight) was administered intraperitoneally in 200 µL 0.9% saline, twice a day for 4 days, repeated weekly for 3 weeks. Nicotinaldehyde (30 mg/kg body weight) was administered intraperitoneally in 200 µL 0.9% saline, once a day for 4 days, repeated weekly for 3 weeks. Control groups were similarly treated but with saline solution. All animals were monitored daily for signs of illness and killed immediately if the tumor size reached a volume of 1000 mm^3^.

### 2.6. Immunoblotting

Protein samples were separated by SDS-PAGE on a 10% polyacrylamide gel and analyzed by immunoblotting. The mouse anti-NAPRT (HPA023739) and the rabbit anti-actin (ab1801) antibodies were purchased from Abcam (Amsterdam, The Netherlands). After incubation with primary antibodies, polyclonal goat anti-mouse IgG conjugated with IRDye 680 (LI-COR, B70920-02, LI-COR Biosociences, Lincoln, NE, USA) or goat anti-rabbit IgG conjugated with IRDye 800 (LI-COR, 926-32210) were applied. Protein bands were visualized using the Odyssey Infrared Imaging System (LI-COR).

### 2.7. Statistical Analyses

Unless specified otherwise, data are expressed as mean ± SD. The experiments were repeated independently at least three times. Statistical analyses were conducted using GraphPad Prism 8.0 Software (GraphPad Software, San Diego, CA, USA). Unpaired t-tests were used for evaluating differences between groups. For animal survival analyses, Kaplan–Meier method using the log rank test was applied. Statistical significance was established for *p* < 0.05.

## 3. Results

### 3.1. Nicotinaldehyde Abrogates the Anti-Leukemic Effect of APO866

**Figure 1:**
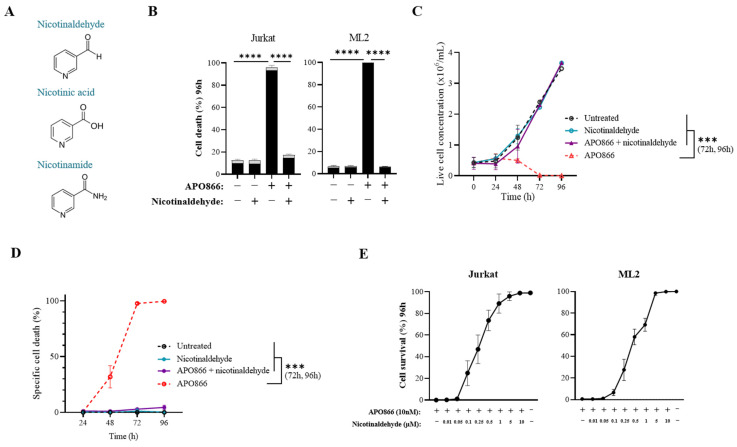
Nicotinaldehyde supplementation abrogates APO866 cytotoxicity in leukemia cells. (**A**) Chemical structures of nicotinaldehyde (3-pyridinecarboxaldehyde) and of NAD precursors nicotinic acid and nicotinamide. (**B**) Cell death of Jurkat and ML2 cells at 96 h of APO866 (10 nM) treatment with or without 10 µM of nicotinaldehyde supplementation (black box = 7AAD^+^, late apoptosis and necrosis. Grey box = annexin V^+^ 7AAD^−^, early apoptosis). (**C**) Time course cell growth evaluated from live cell number and (**D**) time course cell death rate of Jurkat cells treated with APO866 (10 nM) for 96 h, with or without 10 µM nicotinaldehyde supplementation. (**E**) Cell survival of Jurkat and ML2 cells treated with APO866 (at 10 nM) and nicotinaldehyde at concentrations ranging from 0.01 to 10 µM for 96 h. Error bars represent SD of at least 3 independent replicates. *** *p* < 0.001, **** *p* < 0.0001.

Recently, we showed that bacteria present in the cell culture or in gut microbiota can significantly lower the antitumor efficacy of APO866 through the catalytic activity of nicotinamidase that converts NAM into NA, thus fueling an alternative NAD synthesis pathway [16]. In continuation with our previous study, here we used nicotinaldehyde (or 3-pyridinecarboxaldehyde), known as a potent inhibitor of nicotinamidase [18,19] (Figure 1A), with the attempt to reverse the bacterial protective effect in APO866-treated malignant cells. Surprisingly, nicotinaldehyde treatment did not resensitize mycoplasma-infected leukemia cells to APO866, but rather abrogated APO866-antitumor activity in uninfected cells (Figure S2).

Nicotinaldehyde is the aldehyde form of the well-described NAD precursor NA, however, the implication of nicotinaldehyde in the NAD metabolome in mammalian cells has not been explored so far. To evaluate the potential effect of nicotinaldehyde on the antitumor activity of APO866, we treated two human leukemia cell lines, Jurkat and ML2 cells, with APO866 at 10 nM for 96 h without or with nicotinaldehyde (10 µM) and monitored the cell viability. As shown in Figure 1, the supplementation of nicotinaldehyde did not affect cell viability (Figure 1B) nor proliferative capacity (Figure 1C), indicating that this dose of nicotinaldehyde is well tolerated by leukemia cells. While treatment with APO866 killed 100% of leukemia cells, co-treatment with nicotinaldehyde allowed cells to survive and proliferate at the same rate as the untreated cells (Figure 1B,C). Accordingly, we showed that nicotinaldehyde is able to abrogate cell death induction by NAMPT inhibition. A time course analysis of cell death rate demonstrated that nicotinaldehyde effectively protects leukemia cells from the APO866 cytotoxic effect from the early phase of the treatment (Figure 1D). We also showed that the supplementation with nicotinaldehyde conferred a dose-dependent protection in APO866-treated leukemia cells (Figure 1E). The half maximal effective concentration, defined as the concentration of nicotinaldehyde that reverses 50% of APO866-induced cell death, was estimated to be 0.47 µM in both Jurkat and ML2 cells. To further extend these observations, the same analysis was performed on additional hematologic malignant cell lines. Consistent with our initial finding, the supplementation with nicotinaldehyde abrogated the antitumor activity of APO866 on all tested cell lines (Figure S3).

Overall, the results indicate that nicotinaldehyde supplementation protects leukemia cells from APO866-induced cytotoxicity.

### 3.2. Nicotinaldehyde Prevents APO866-Induced Intracellular NAD Depletion, Subsequent Oxidative Stress, Mitochondrial Membrane Depolarization and ATP Depletion

**Figure 2:**
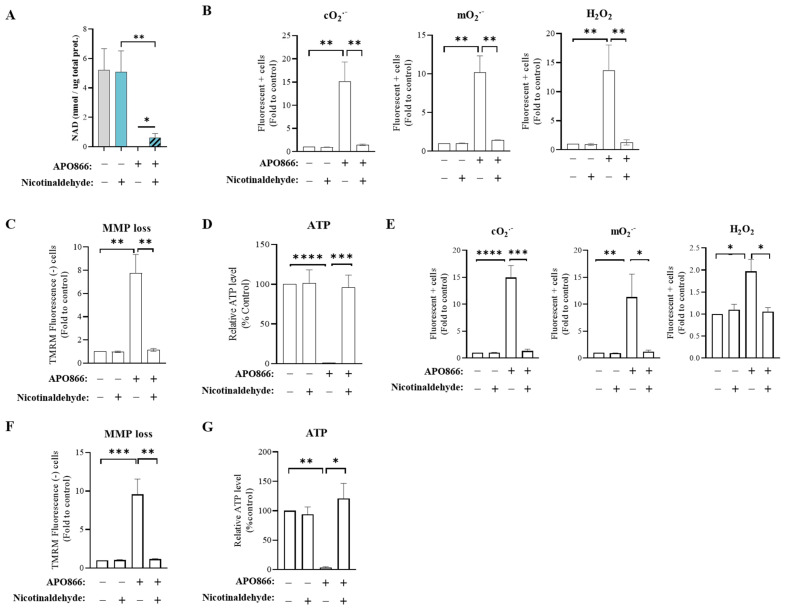
Nicotinaldehyde supplementation counteracts NAD depletion and abrogates cytotoxic events induced by APO866. Jurkat and ML2 cells were treated without or with 10 nM of APO866 in absence or presence of 10 µM of nicotinaldehyde supplementation for 96 h to assess various cytotoxic signatures of APO866. (**A**) Intracellular total NAD (NAD^+^ and NADH) measured by biochemical assay and normalized to protein concentration, in Jurkat cells at 96 h of treatment. (**B**) Intracellular ROS (cytosolic and mitochondrial superoxide O_2_^−^ and cellular hydrogen peroxide H_2_O_2_) levels measured with specific probes (DHE, mitosox and H2DCFDA, respectively) by flow cytometry in Jurkat cells. (**C**) Mitochondrial membrane potential (MMP) determined with TMRM staining, using flow cytometry in Jurkat cells (**D**) ATP concentration measured by luminescence assay, relative to the concentration in untreated cells. (**E**) ROS, (**F**) MMP and (**G**) ATP concentration in ML2 cells. Data are mean ± SD, n ≥ 3. * *p* < 0.05, ** *p* < 0.01, *** *p* < 0.001, **** *p* < 0.0001.

The primary effect of APO866, causing subsequent cancer cell death, is the depletion of the intracellular NAD content (by approximately >95%) [20]. Therefore, we next evaluated the intracellular levels of NAD in APO866-treated leukemia cells in the absence or presence of nicotinaldehyde. As shown in Figure 2A and in line with previous studies, APO866 fully depleted intracellular NAD content in Jurkat cells. Contrastingly, APO866-treated leukemia cells that received nicotinaldehyde supplementation showed partial but significant maintenance of intracellular NAD at approximately 13% of the amount in untreated control cells. These observations suggest that nicotinaldehyde enables the replenishment of intracellular NAD despite the inhibition of NAMPT, indicating that nicotinaldehyde could be a potential novel source of NAD biosynthesis. Supplementation of nicotinaldehyde alone had no effect on the intracellular NAD level, suggesting that leukemia cells already had enough substrates and that their NAD levels were in equilibrium. This observation is consistent with the global notion that the intracellular NAD metabolome is tightly regulated in the cells. Additionally, we tested whether nicotinaldehyde can still protect leukemia cells from APO866-induced cell death if it is supplemented only when NAD is already depleted by APO866. We first treated leukemia cells with APO866 (10 nM) alone, and at 24 h after drug treatment, nicotinaldehyde (10 µM) was supplemented and the cells were further incubated for an additional 72 h. As shown in Figure S4A, nicotinaldehyde was able to protect leukemia cells from APO866-induced cell death, although NAD depletion was already induced at 24 h when nicotinaldehyde was added to the cell cultures (Figure S4B). These observations suggest that nicotinaldehyde can be rapidly exploited by leukemia cells to replenish intracellular NAD and inhibit APO866-induced cell death.

The cytotoxic events involved in the tumor-killing activity of APO866 have been well described in a previous study [21]. Notably, NAD depletion induced by APO866 triggers oxidative stress with the generation of high levels of reactive oxygen species (ROS), mitochondrial membrane depolarization and subsequent ATP depletion. Next, we investigated whether nicotinaldehyde supplementation prevents these cytotoxic events in APO866-treated leukemia cells. As reported in Figure 2B, we observed that treatment with APO866 alone significantly increased intracellular ROS levels in Jurkat cells: cytosolic and mitochondrial superoxides increased, respectively, by 15- and 13-fold compared to the untreated conditions, and hydrogen peroxide increased 13-fold. Mitochondrial membrane depolarization was enhanced 7-fold compared to the control (Figure 2C). Intracellular ATP dropped to an undetectable level with APO866 treatment (Figure 2D). Supplementation with nicotinaldehyde maintained intracellular ROS, mitochondrial membrane potential and ATP content at control levels (Figure 2B–D). Similarly, nicotinaldehyde abrogated these events in ML2 cells (Figure 2E–G). Collectively, the data demonstrate that nicotinaldehyde supplementation protects leukemia cells from the subsequent cytotoxic events induced by NAD depletion upon treatment with APO866.

### 3.3. Nicotinaldehyde Protective Function Requires the Integrity of NAPRT

**Figure 3:**
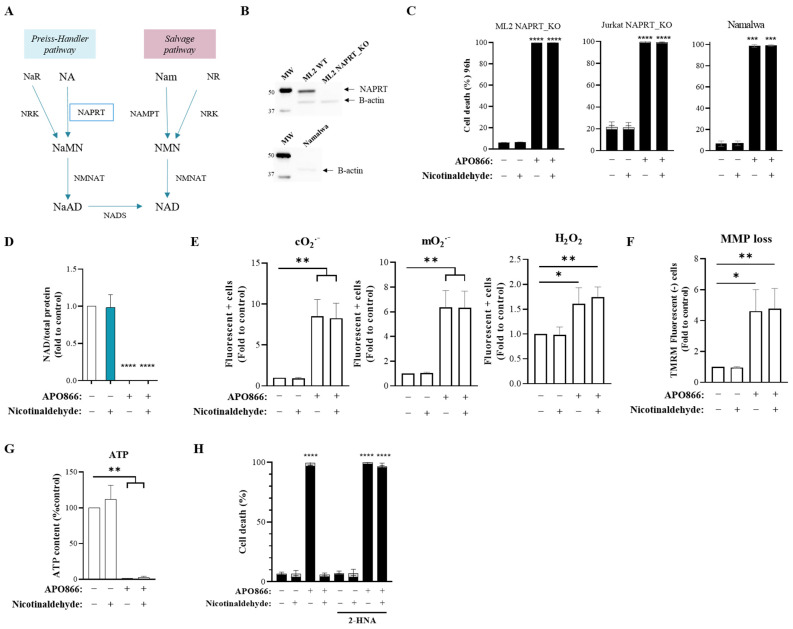
The nicotinaldehyde protective effect is dependent on NAPRT. (**A**) Schematic representation of major NAD biosynthetic pathways. Nam, nicotinamide; NR, nicotinamide riboside; NMN, Nam mononucleotide; NAMPT, Nam phosphoribosyltransferase; NRK, NR kinase; NMNAT, NMN adenylyltransferase; NA, nicotinic acid; NaR, nicotinic acid riboside; NaMN, NA mononucleotide; NAPRT, NA phosphoribosyltransferase; NADK, NAD kinase. (**B**) Western Blot images of NAPRT protein in ML2 WT, ML2 NAPRT KO and Namalwa cells. MW, molecular weight. (**C**) Cell death (black box = 7AAD^+^, late apoptosis and necrosis. Grey box = annexin V^+^ 7AAD^−^, early apoptosis) of NAPRT-lacking Jurkat NAPRT KO, ML2 NAPRT KO and Namalwa cells at 96 h of APO866 10 nM treatment. Uncropped Western Blots are available in Figure S7 (**D**) Intracellular NAD level in Jurkat NAPRT KO cells treated without or with APO866 (10 nM) in absence or presence of nicotinaldehyde supplementation (10 µM) for 96 h. (**E**) Intracellular levels of ROS (cytosolic and mitochondrial O_2_^.−^ and cellular H_2_O_2_ measured with specific probes (DHE, mitosox and H2DCFDA). (**F**) MMP determined using flow cytometry, after cell staining with TMRM. (**G**) ATP concentration measured by luminescence assay and normalized to the protein concentration in untreated cells at the corresponding time point. (**H**) Cell death at 96 h of ML2 cells treated without or with APO866 and nicotinaldehyde, in the absence or presence of 2 mM 2-hydroxynicotinic acid (2-HNA). Data are mean ± SD, n ≥ 3. * *p* < 0.05, ** *p* < 0.01, *** *p* < 0.001, **** *p* < 0.0001.

Next, we sought to investigate how nicotinaldehyde contributes to the maintenance of intracellular NAD. The oxidation of aldehydes to their related carboxylic acids is a well-known phenomenon. This conversion can occur either via the action of enzymes with aldehyde oxidizing and reducing activities or spontaneously in the presence of oxygen [22,23]. Based on this notion, we hypothesized that nicotinaldehyde can be converted to NA either spontaneously or enzymatically. In this scenario, there might be an involvement of NAPRT, the rate-limiting enzyme of the NAD biosynthesis pathway that utilizes NA as the starting substrate (Figure 3A), in the replenishment of intracellular NAD from nicotinaldehyde. To test this hypothesis, we used cancer cells that were either endogenously deficient in functional NAPRT (Namalwa) (Figure 3B) or that lacked the NAPRT gene by genetic knock-out (Jurkat NAPRT KO and ML2 NAPRT KO). As shown in Figure 3C, the measurement of cell death in APO866 and nicotinaldehyde-co-treated Jurkat NAPRT KO, ML2 NAPRT KO and Namalwa cells revealed that nicotinaldehyde could not protect these cells from APO866-induced cell death. We next evaluated the impact of nicotinaldehyde supplementation on intracellular metabolites and ROS levels in these cells. The measurement of intracellular NAD level in Jurkat NAPRT KO cells showed that nicotinaldehyde supplementation failed to counteract the depletion of NAD upon treatment with APO866 (Figure 3D). Consequently, the cytotoxic events induced by APO866 were not prevented, as illustrated by the increase of ROS, the mitochondrial membrane depolarization and the depletion of ATP (Figure 3E–G). In a similar manner, in Namalwa cells nicotinaldehyde supplementation could not counteract APO866-induced cytotoxic events (Figure S5). Additionally, in order to assess whether the catalytic function of NAPRT is essential, we tested the effect of the potent NAPRT inhibitor 2-hydroxynicotinic acid (2-HNA) on the nicotinaldehyde tumor-protective function against APO866. Similar to NAPRT genetic knock-out, the chemical inhibition of NAPRT with 2-HNA (2 mM) abrogated the protection by nicotinaldehyde from APO866-induced cell death (Figure 3H). Given that 2-HNA acts as a competitive inhibitor of the enzymatic binding pocket of NAPRT [24,25], the protective function of nicotinaldehyde seems to mediate the enzymatic activity of NAPRT. Altogether, the data demonstrate that the integrity of NAPRT status is essential for the replenishment of NAD, prevention of oxidative stress, ATP depletion, and consequent rescue from APO866-induced cell death with nicotinaldehyde supplementation.

### 3.4. Nicotinaldehyde Boosts the Level of NA and Activates the Preiss–Handler Pathway as an Alternative Route of NAD Biosynthesis to Circumvent the Anti-Leukemic Activity of APO866

**Figure 4:**
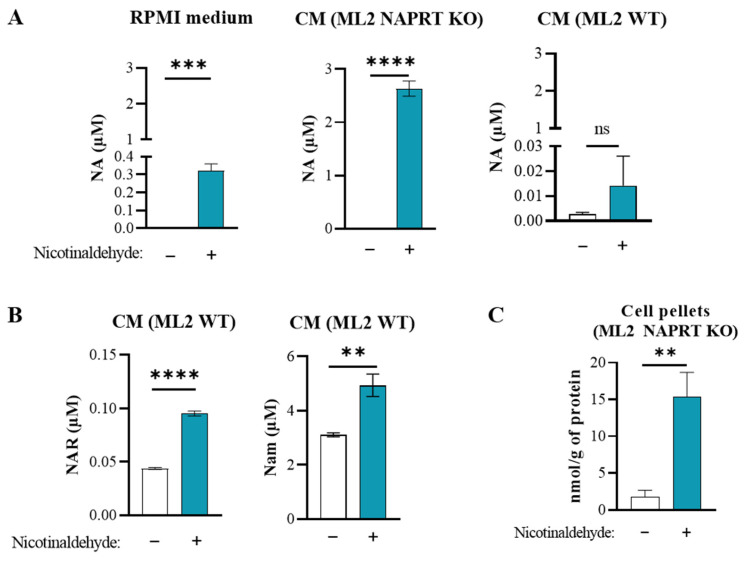
Nicotinaldehyde supplementation modifies NAD-related metabolite levels in the culture medium and within cells. NAD metabolites were quantified by LC-MS/MS in cells and conditioned media (CM) after 96 h of incubation at 37 °C. (**A)** NA concentration in the culture medium supplemented without (−) or with (+) nicotinaldehyde (10 µM), in absence of cells or in presence of ML2 WT or ML2 NAPRT KO cells, after 96 h of incubation at 37 °C. (**B**) NAM and NAR concentrations in the conditioned media of ML2 WT cells without or with nicotinaldehyde supplementation (10 µM). (**C**) Intracellular concentration of NA normalized to protein concentration in ML2 NAPRT KO cells cultured in medium supplemented without or with nicotinaldehyde (10 µM). n = 3 error bars = SD, ns = non-significant. ** *p* < 0.01, *** *p* < 0.001, **** *p* < 0.0001 (vs. not supplemented medium if not specified otherwise).

To provide evidence of the conversion of nicotinaldehyde to NA, we next investigated whether a spontaneous oxidation of nicotinaldehyde to NA can occur in RPMI culture medium supplemented with nicotinaldehyde. To this end, we quantified the NAD-related metabolites by LC-MS/MS in RPMI medium supplemented or not with 10 µM of nicotinaldehyde. As reported in Figure 4A, the supplementation with nicotinaldehyde yielded a significant increase of NA from 0.003 µM to 0.3 µM in RPMI culture medium, suggesting a spontaneous oxidation of nicotinaldehyde to NA. No significant variation in any other NAD metabolites was observed (Figure S6A). Secondly, mammalian cells possess multiple enzymes that are capable of catalyzing the oxidation of aldehydes to their corresponding carboxylic acids [22]. This notion suggests that these enzymes in leukemia cells can significantly contribute to the conversion of nicotinaldehyde to NA. To test this hypothesis, we quantified NAD metabolites in the conditioned media (CM) of ML2 NAPRT KO cells cultured in the absence or presence of nicotinaldehyde. As ML2 NAPRT KO cells are not able to consume NA, the extent of conversion can be expected to be strongly evidenced when compared to that in ML2 WT cells, which are potentially able to consume the generated NA. In accordance with this hypothesis, Figure 4A shows that the CM of ML2 NAPRT KO cells supplemented with nicotinaldehyde displayed a tremendous increase of NA (2.8 µM) when compared with the CM without supplementation (0.003 µM). The amount of converted NA upon supplementation in the presence of leukemia cells corresponds to a significant increase that is almost 10 times higher than in the absence of cells (0.28 µM). No significant variation of any other metabolite was observed (Figure S6B). With ML2 WT cells, supplementation with nicotinaldehyde resulted in a higher level of extracellular NA in the CM compared to control (from 0.015 µM to 0.003 µM, non-significant change) (Figure 4A), but to a much less extent when compared with the increase with ML2 NAPRT KO cells (2.8 µM), indicating that the generated NA is presumably consumed by ML2 WT cells. In addition, in the CM of ML2 WT cells, supplementation with nicotinaldehyde led to high levels of other NAD precursors including NaR (0.095 µM compared to 0.044 µM), and NAM (4.9 µM compared to 3.1 µM) (Figure 4B, Figure S6C). Moreover, we measured the NAD metabolites in the cell pellets of ML2 cells cultured without or with nicotinaldehyde supplementation and observed that intracellular NA in ML2 NAPRT KO cells was also increased by 6-fold (Figure 4C, Figure S6D). This indicates that considerable amounts of nicotinaldehyde have been transformed to NA and uptaken within cells. We did not observe any other significant variations in the intracellular metabolite levels within the ML2 WT cells (Figure S6E), consistent with the notion of a tight regulation of NAD metabolites levels in the cells with sufficient substrates. Of note, nicotinaldehyde supplementation did not affect the levels of any metabolite of the de novo pathway.

Collectively, our observations indicate that the supplemented nicotinaldehyde is converted to NA in a cell-dependent manner and is used as an alternative precursor for NAD biosynthesis through the NAPRT-dependent Preiss–Handler pathway.

### 3.5. Administration of Nicotinaldehyde Blunts the Antitumor Activity of APO866 in Mouse Xenograft Model of Human Leukemia

**Figure 5:**
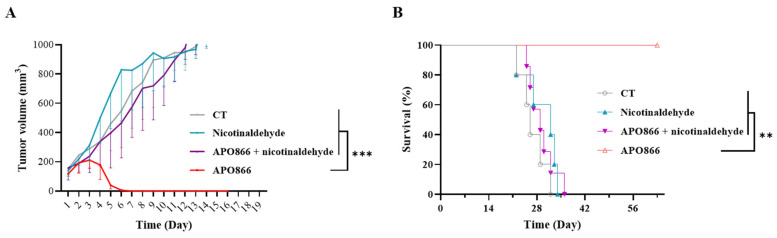
Nicotinaldehyde abrogates APO866 antitumor effect in a mouse xenograft model of human leukemia. (**A**) Evolution of tumor growth and (**B)** survival rate of mice xenografted with ML2 cells and treated without or with APO866 in absence or presence of nicotinaldehyde. Control group received vehicle (grey line). Nicotinaldehyde (30 mg/kg; blue line), APO866 (10 mg/kg; red line) or nicotinaldehyde plus APO866 (purple line) were administered as described in “Materials and Methods”. Day 1 corresponds to the beginning of the treatment (when tumor volume reached >100mm^3^) (**A**) or the day of tumor injection (**B**). n = 5 per group, error bars = SD. ** *p* < 0.01, *** *p* < 0.001. Data were analyzed by Kaplan–Meier survival analysis with log-rank test.

Next, we evaluated the impact of nicotinaldehyde on the therapeutic efficacy of APO866 in vivo using a mouse xenograft model of human leukemia. To this end, we subcutaneously injected ML2 cells in SCID mice. Once the tumor appeared and reached a volume of 100–150 mm^3^, mice were randomized into control and treated groups. Mice were administered intraperitoneally with (i) saline solution (control); (ii) APO866 (15 mg/kg); (iii) nicotinaldehyde (30 mg/kg); and (iv) APO866 + nicotinaldehyde. Of note, we did not observe any signs of toxicity in all mice, including those that were treated with nicotinaldehyde, indicating that nicotinaldehyde treatment was safe at the chosen dose. Without any treatment (control group), or with nicotinaldehyde treatment alone, tumors grew rapidly and reached the endpoint volume of 1000 mm^3^ within two weeks after tumor appearance (Figure 5A). APO866 treatment eradicated tumor growth within one week. However, when mice were co-treated with APO866 and nicotinaldehyde, the tumor growth was comparable to that of the control group, demonstrating that nicotinaldehyde fully blunted the APO866 antitumor effect. This led to overall survival rates that are comparable between the control, nicotinaldehyde-administered and nicotinaldehyde + APO866 co-treated groups, where all mice reached the endpoint within four weeks after tumor injection (Figure 5B). Contrastingly, APO866-administered mice survived for more than eight weeks of observation after tumor injection and remained disease-free without any appearance of a palpable tumor. Taken together, these results in a mouse xenograft model demonstrate that nicotinaldehyde abrogates in vivo the therapeutic efficacy of APO866.

## 4. Discussion

Here, we report a previously undescribed function of nicotinaldehyde in NAD metabolism and its negative impact on the therapeutic efficacy of NAMPT inhibitors in leukemia treatment. We demonstrated that nicotinaldehyde can be used as a novel NAD precursor by leukemia cells to circumvent the NAD depletion typically induced by NAMPT inhibition blocking the NAD salvage pathway. Mechanistically, our data demonstrate that when present in a tumor environment, nicotinaldehyde is converted to NA either spontaneously or in a cell-dependent manner. NA converted from nicotinaldehyde is exploited through the NAPRT-dependent Preiss–Handler pathway. Although nicotinaldehyde supplementation only partially restores intracellular NAD decreased by APO866 in leukemia cells, the residual NAD synthesized from the converted NA is sufficient to prevent ROS production, preserve mitochondrial integrity, and abrogate ATP depletion and thus protects leukemia cells from APO866-induced cell death. Furthermore, using a xenograft model of human leukemia, we provide evidence that nicotinaldehyde can severely hamper the in vivo efficacy of APO866.

Very few studies on nicotinaldehyde are reported in the scientific literature. Nicotinaldehyde is a natural compound found in plants and bacteria, which can also be contained in foods of plant origin such as honey [17]. However, little is known about the role of nicotinaldehyde in the NAD metabolism in humans. A previous study by Shibata et al. [26] demonstrated that administering nicotinaldehyde in an NA-free Tryptophan-limiting diet to rats can significantly boost the NAD level in the blood and liver. These authors also showed that nicotinaldehyde can be used as a precursor of NAD with the same efficiency as NA. Our study supports this previous report and complements it by demonstrating that nicotinaldehyde is exploited through conversion to NA and further transformation in the Preiss–Handler pathway.

The conversion of nicotinaldehyde to NA is presumably catalyzed by enzyme(s) with aldehyde oxidative activity. Several enzymes such as aldo-keto reductases, alcohol dehydrogenase (ADH), or aldehyde dehydrogenase (ALDH) demonstrated catalytic activities toward nicotinaldehyde in vitro [27,28,29]. Although we sought to identify the responsible enzymes for this conversion, we have not obtained conclusive results so far. The precise mechanism remains to be established and is an objective of future research in the laboratory.

The data also suggest that nicotinaldehyde can be administrated safely in animals at the chosen doses as demonstrated by the absence of any signs of toxicity such as loss of body weight, lethargy, rough coat and no premature deaths. The safety of nicotinaldehyde could be most probably explained by the existence, in mice as well as in humans, of a large number of aldehyde metabolizing enzymes, as mentioned above. In line with this assumption, several reported studies in which rats were fed diets supplemented with nicotinaldehyde have been carried out without toxicity while showing significant activities comparable to NA at the same doses [26,30].

It is noteworthy to mention that nicotinaldehyde is mostly reported as a potent inhibitor of nicotinamidase, an enzyme that is absent in mammals but widely distributed in several species of bacteria, plants and many metazoan species [18]. This enzyme converts NAM to NA, and its importance in mammalian systemic NAD homeostasis has emerged recently through several studies showing the implication of gut microbial enzymes in the modulation of host NAD metabolite levels [15,16,31]. Nicotinaldehyde could therefore be considered to block the conversion of NAM to NA and its subsequent use through the NAPRT pathway. However, our results strongly suggest that nicotinaldehyde can act as an alternative precursor for NAD biosynthesis, rather than blocking the above-mentioned biosynthetic route through nicotinamidase. This opposite function should be considered carefully when using nicotinaldehyde as an inhibitor of nicotinamidase.

Importantly, although our data revealed a negative impact of a novel NAD precursor on the anti-leukemic activity of APO866, we also demonstrated that the sensitivity of leukemia cells can be restored by genetically silencing or pharmacologically inhibiting NAPRT. These results support the strategy of a dual targeting of the two major NAD biosynthesis pathways to induce robust NAD depletion and anti-cancer therapeutic efficacy, despite the potential interference of NAD-related metabolites present in the tumor environment.

Our metabolomic analysis revealed a significant increase of NaR in the conditioned medium of ML2 WT cells upon supplementation with nicotinaldehyde. A study from Kulikova et al. [32] showed that NaR can be generated from NA via NaMN formation (by NAPRT) and its dephosphorylation by cytosolic 5′-nucleotidases (NT5), under conditions that lead to the excessive formation of NaMN. We can speculate that the successive conversions of nicotinaldehyde to NA, and NA to NAMN, may have led to the observed increase of NaR released in the culture medium through a similar mechanism via NT5. Although additional investigation is needed to confirm this hypothesis, this observation illustrates the complex network and inter-conversions of NAD precursors, and the potential importance of riboside NAD precursors, which are worth exploring further.

## 5. Conclusions

These findings identify nicotinaldehyde as a novel member of the NAD metabolome that can contribute to NAD biosynthesis and modulate the response to NAMPT inhibition in leukemia cells. Our study suggests the complexity of the NAD metabolome and therefore stresses the need to further explore the interactions between NAD precursors. The results highlight the crucial role of the NAPRT-dependent pathway in cell survival and demonstrate the importance of targeting both NAMPT- and NAPRT-dependent pathways in order to optimize the therapeutic efficacy of NAMPT inhibitors.

## Data Availability

The data presented in this study are available on request from the corresponding author.

## Supplementary Materials

The following supporting information can be downloaded at: https://www.mdpi.com/article/10.3390/cancers15030787/s1, Figure S1: CD38 expression status of ML2, Jurkat and Namalwa cells; Figure S2: Nicotinaldehyde does not resensitize mycoplasma-infected leukemia cells to APO866, but rather abrogates APO866-antitumor activity in uninfected cells; Figure S3: Nicotinaldehyde supplementation abrogates APO866 cytotoxicity in several hematological cancer cell lines; Figure S4: Nicotinaldehyde supplementation at 24 h of APO866 treatment rescues ML2 and Jurkat cells from APO866-induced cell death; Figure S5: Nicotinaldehyde supplementation does not counteract cytotoxic events induced by APO866 in Namalwa cells; Figure S6: NAD-related metabolite levels in the culture medium, cells and conditioned media upon nicotinaldehyde supplementation; Figure S7: Original Western Blot for Figure 3C.
