# Supplementary material for: Nicotinaldehyde, a Novel Precursor of NAD Biosynthesis, Abrogates the Anti-Cancer Activity of an NAD-Lowering Agent in Leukemia
Source: Cancers (Basel). 2023 Jan 27;15(3):787. doi: 10.3390/cancers15030787 (PMC9913462; doi:10.3390/cancers15030787)
Supplement: Supplementary file 1 [file cancers-15-00787-s001.zip › cancers-2067013-supplementary.pdf]

cancers

MDPI

Supplementary Materials: Nicotinaldehyde, a Novel Precursor of NAD Biosynthesis, Abrogates the Anti-Cancer Activity of an NAD-Lowering Agent in Leukemia

Saki Matsumoto, Paulina Biniecka, Axel Bellotti, Michel A Duchosal and Aimable Nahimana

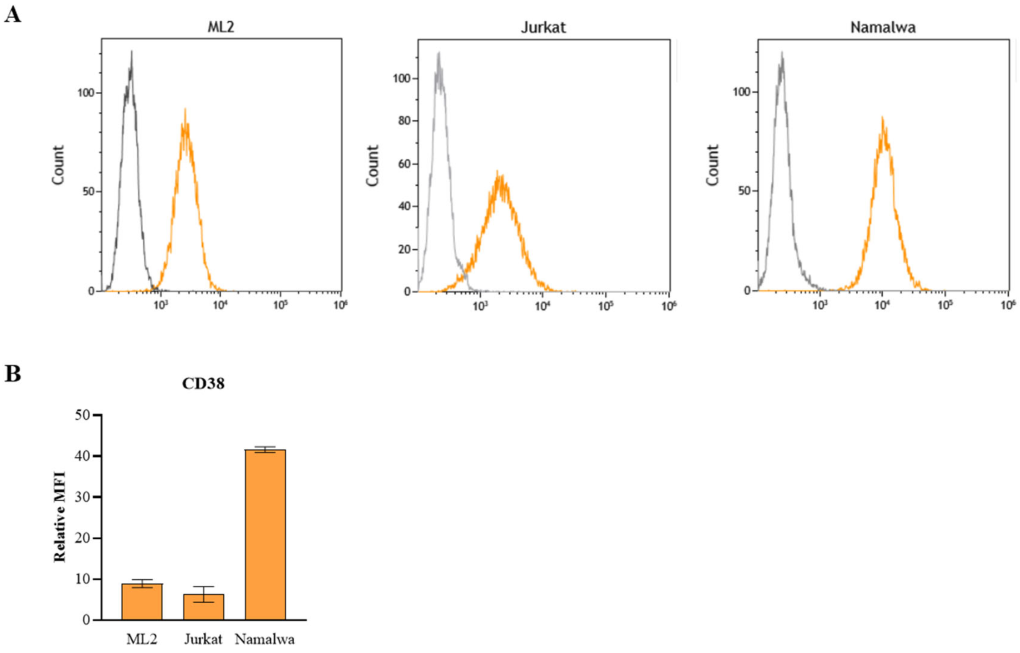

Figure S1. CD38 expression status of ML2, Jurkat and Namalwa cells.CD38 expression was evaluated in ML2, Jurkat and Namalwa cells by flow cytometry. (**A**) Representative fluorecence levels of cells labelled with CD38-specific (orange) and matched isotype control-targeted (grey) antibodies. (**B**) Relative fluorecence intensities calculated as the ratio of mean fluorescence intensities (MFI) of cells labelled with CD38-specific antibody and the control. Data are mean ± SD, n = 3.

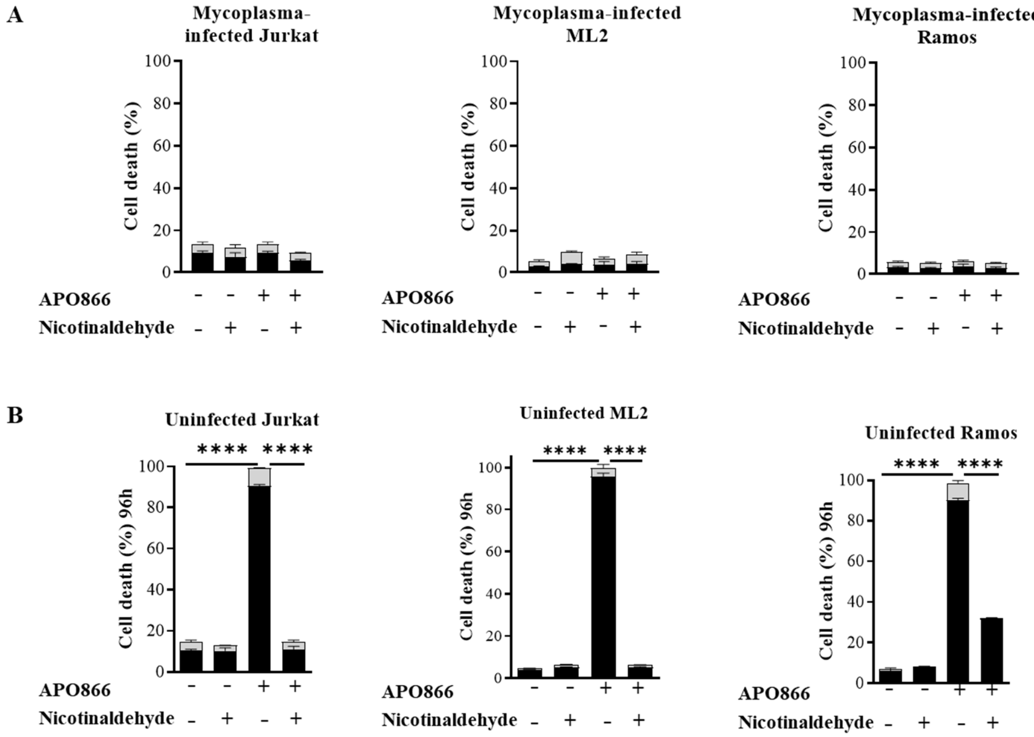

Figure S2. Nicotinaldehyde does not resensitize mycoplasma-infected leukemia cells to APO866, but rather abrogates APO866-antitumor activity in uninfected cells. ML2, Jurkat and Ramos cells were infected (**A**) or unifected (**B**) with mycoplasma as described previously [16]. Cell death was assessed at 96 h of APO866 (10 nM) treatment with or without 250 µM of nicotinaldehyde supplementation (black box = 7AAD+, late apoptosis and necrosis. Grey box = annexin V+ 7AAD− , early apoptosis). Data are mean ± SD, $n = 3$. **** $p < 0.0001$.

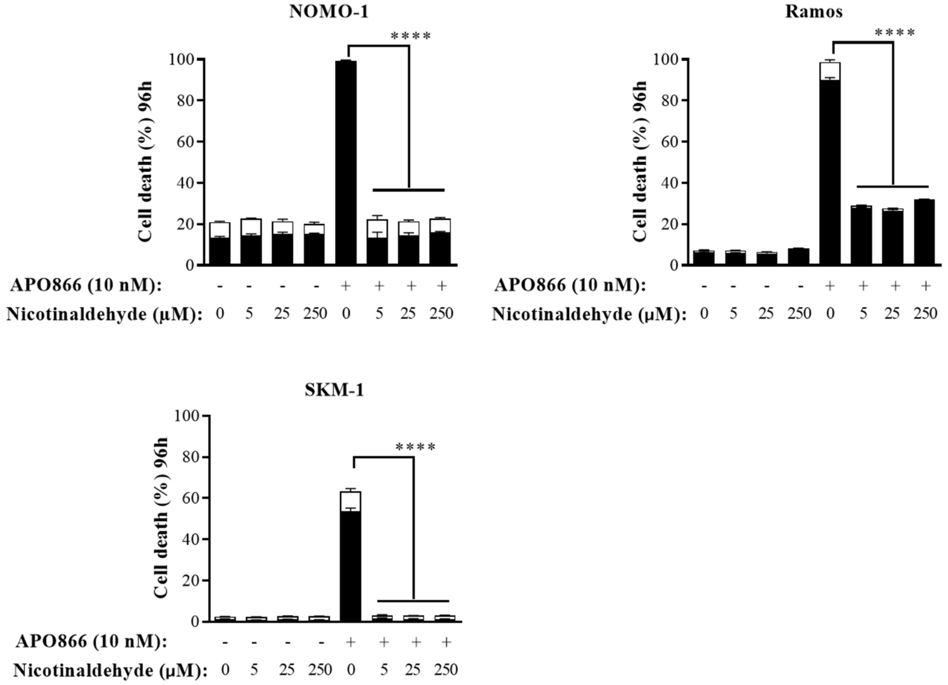

Figure S3. Nicotinaldehyde supplementation abrogates APO866 cytotoxicity in several hematological cancer cell lines. Cell death of Ramos (Burkitt lymphoma), SKM-1 and NOMO-1 (acute myeloid leukemia) cells assessed at 96 h of APO866 treatment (10nM) with or without nicotinaldehyde supplementation at 5, 25 and 250 μM. Data are mean ± SD, $n = 3$. **** $P < 0.0001$.

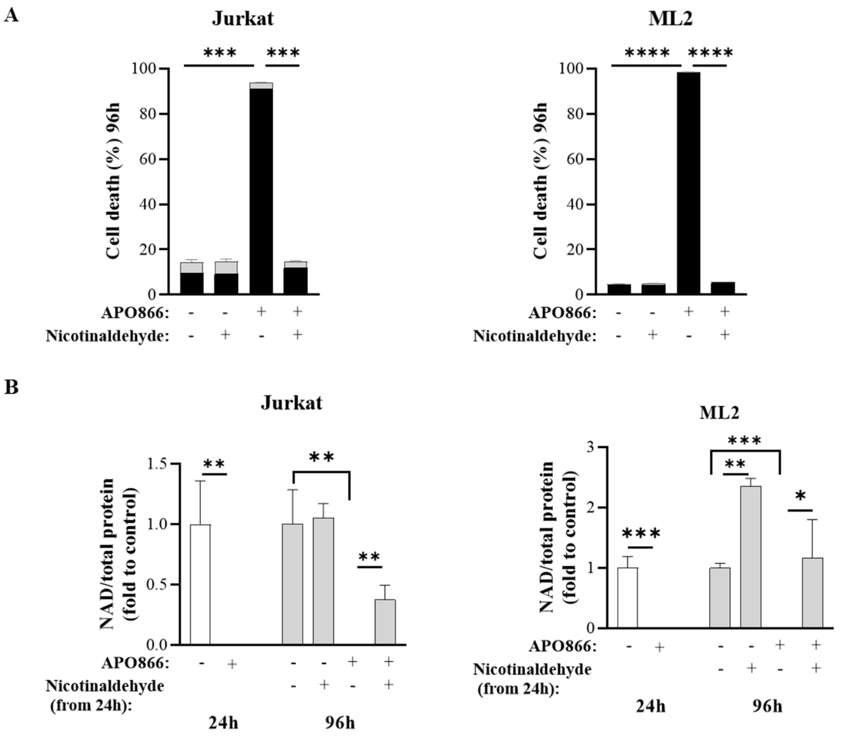

Figure S4. Nicotinaldehyde supplementation at 24 h of APO866 treatment rescues ML2 and Jurkat cells from APO866-induced cell death. ML2 and Jurkat cells were treated with APO866 (10 nM) and after 24 h of treatment, 10 μM of nicotinaldehyde was added to cell cultures that were further incubated for additional 72 h. (**A**) Cell death assessed at 96 h of APO866 treatment (black box = 7AAD+, late apoptosis and necrosis. Grey box = annexin V+ 7AAD− , early apoptosis). (**B**) Intracellular NAD levels evaluated at 24 h (before addition of nicotinaldehyde) and at 96 h. Data are mean ± SD, $n = 3$. * $P < 0.05$, ** $P < 0.01$, *** $P < 0.001$, **** $P < 0.0001$.

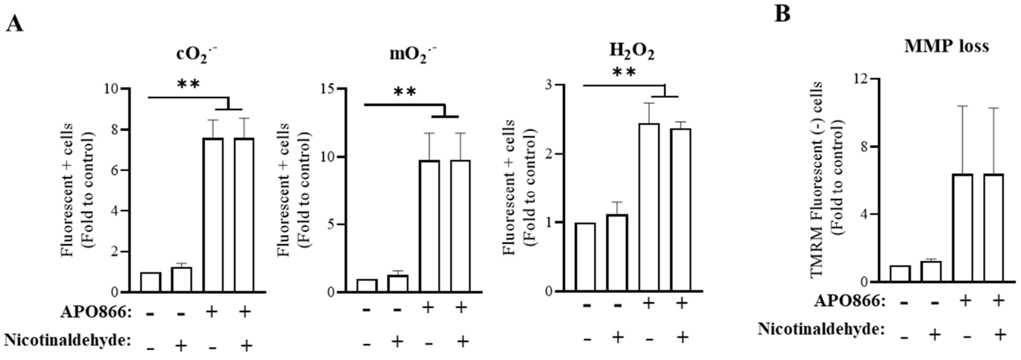

Figure S5. Nicotinaldehyde supplementation does not counteract cytotoxic events induced by APO866 in Namalwa cells. Namalwa cells were treated with APO866 (10 nM) in absence or presence of 10 μM of nicotinaldehyde supplementation and different cytotoxic signatures of APO866 were assessed at 96 h of treatment. (**A**) Intracellular ROS (cytosolic and mitochondrial superoxide O_2^{-} and cellular hydrogen peroxide H_2O_2) levels measured with specific probes (DHE, mitosox and

H2DCFDA respectively) by flow cytometry. (**B**) Mitochondrial membrane potential (MMP) determined with TMRM staining, using flow cytometry. Data are mean ± SD, $n = 3$. ** $P < 0.01$.

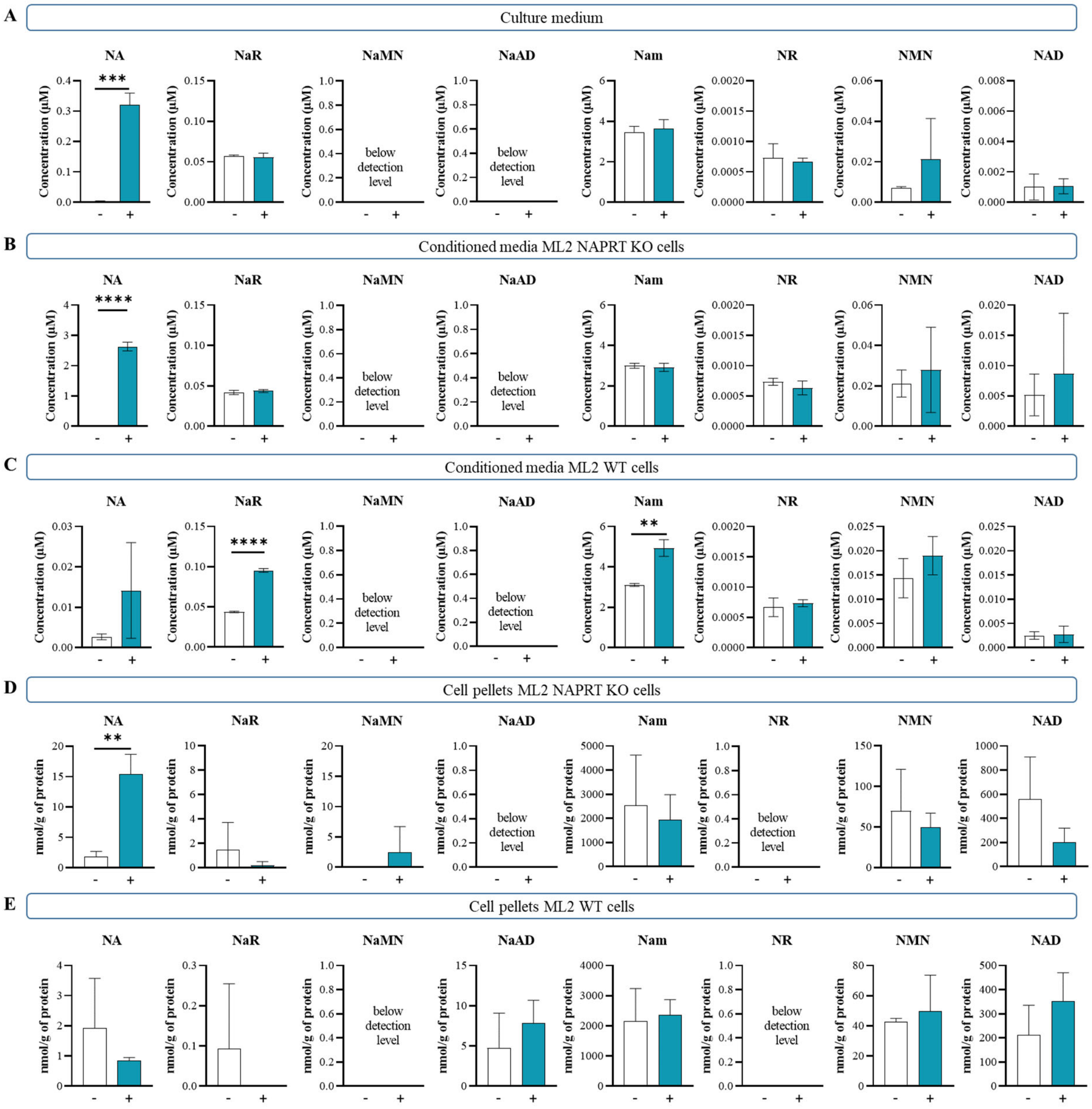

Figure S6. NAD-related metabolite levels in the culture medium, cells and conditioned media upon nicotinaldehyde supplementation. NAD-related metabolite levels were evaluated by LC-MS/MS without or with nicotinaldehyde supplementation (10 µM) and incubation for 96 h in (**A**) the RPMI culture medium (without cells), (**B**) conditioned media of ML2 NAPRT KO cells, (**C**) conditioned media of ML2 WT cells, **D**) cell pellets of ML2 NAPRT KO cells, and **E**) cell pellets of ML2 WT cells. Nam, nicotinamide; NR, nicotinamide riboside; NMN, Nam mononucleotide; NA, nicotinic acid; NaR, nicotinic acid riboside; NaMN, NA mononucleotide; NaAD, NA adenine dinucleotide. Data are mean ± SD, $n = 3$. ** $P < 0.01$, *** $P < 0.001$, **** $P < 0.0001$.

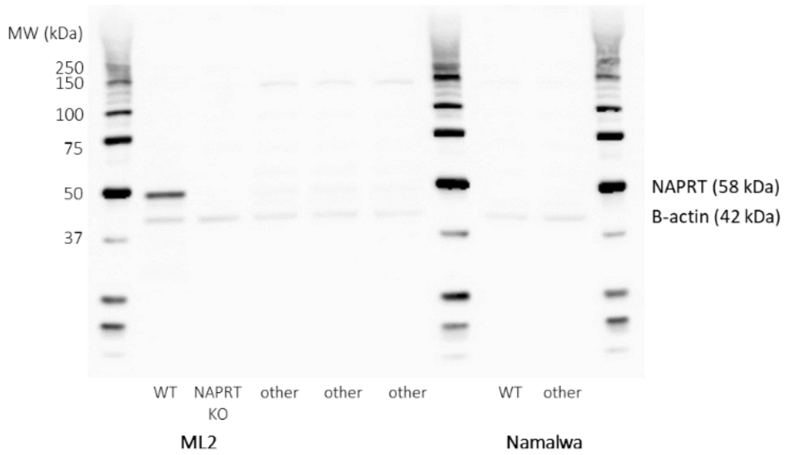

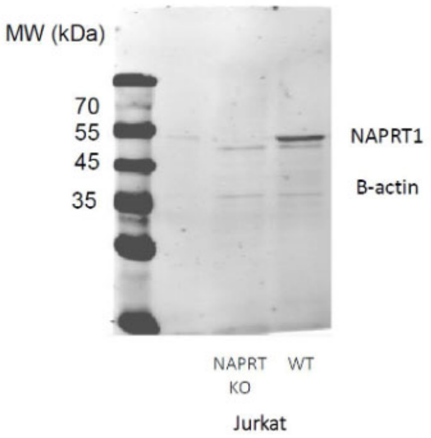

Figure S7. Original Western Blot for Figure 3C.
